# Performance and Synergistic Mechanism of FeSiBCuNb Amorphous Alloy Catalyst for Methylene Blue Degradation

**DOI:** 10.3390/molecules31101720

**Published:** 2026-05-19

**Authors:** Kun Zhang, Feilong Guo, Li Ma, Bin Yu, Tiejun Kuang

**Affiliations:** 1 Tashan Coal Mine, Jinneng Holding Coal Industry Group, Datong 037031, China; 2 College of Resources and Safety, Chongqing University, Chongqing 400030, China; 3 School of Emergency Management and Safety Engineering, China University of Mining and Technology-Beijing, Beijing 100083, China

**Keywords:** amorphous alloy, methylene blue, catalytic degradation, Cu/Nb synergy, cyclic stability

## Abstract

The massive discharge of methylene blue causes severe water pollution, and the development of efficient and stable heterogeneous Fenton catalysts is crucial for wastewater treatment. To address the shortcomings of traditional iron-based amorphous catalysts, such as low activity and poor stability, this study employed Fe_80_Si_6_B_10_Cu_1_Nb_3_ five-component amorphous alloy as the catalyst to investigate its catalytic degradation performance, cyclic stability, and catalytic mechanism for MB. Batch experiments, SEM, XRD characterization, and kinetic fitting were combined to carry out the research. The results showed that under the optimal conditions (25 °C, pH = 3, H_2_O_2_ concentration of 5 mM, catalyst dosage of 0.5 g/L), the catalyst could completely degrade methylene blue within 9 min with a reaction rate constant *k*_obs_ of 0.44 min^−1^, and the degradation efficiency showed no obvious attenuation after 20 consecutive cyclic degradation runs. After degradation, slight selective corrosion occurred on the catalyst surface, while the amorphous structure of the matrix remained stable. This study confirms that the Cu/Nb dual synergy improves the catalytic performance and stability, clarifies the relevant catalytic mechanism, and provides theoretical and technical support for the design of high-performance iron-based amorphous catalysts and the treatment of dye-containing wastewater.

## 1. Introduction

With the rapid development of textile, printing and dyeing industries, the extensive use and discharge of synthetic dyes have become one of the main sources of water pollution [1,2]. Among them, methylene blue (MB) is characterized by bright color, strong light resistance, poor biodegradability, and certain toxicity. Long-term accumulation of MB will destroy the aquatic ecological balance, and it may also pose potential hazards to the human nervous and digestive systems through food chain enrichment [3,4]. Therefore, developing efficient, stable, and low-cost MB degradation technologies is of great practical significance for water pollution control and ecological and environmental protection. Advanced oxidation processes (AOPs) have become a research hotspot in the field of wastewater treatment due to their ability to generate highly oxidizing hydroxyl radicals (·OH), which can rapidly destroy the conjugate system of refractory organic pollutants and achieve their mineralization and degradation [5,6]. Among them, the Fenton reaction (Fe^2+^/H_2_O_2_) is widely used in the treatment of dye-containing wastewater due to its mild reaction conditions, simple operation, and low cost [7,8]. However, in the traditional homogeneous Fenton reaction, Fe^2+^ catalysts are prone to produce iron sludge, causing secondary pollution, and are difficult to recover and reuse, which greatly limits their practical engineering application [9,10]. Therefore, the development of efficient, recoverable, and anti-deactivation heterogeneous iron-based catalysts has become the core breakthrough to solve the above problems.

Iron-based amorphous alloys have been extensively studied as heterogeneous Fenton catalysts due to their disordered atomic arrangement, abundant active sites, and large specific surface area [11,12]. However, they still have obvious shortcomings: traditional FeSiB binary/ternary iron-based amorphous catalysts have limited catalytic activity and slow degradation rate, and are prone to excessive corrosion in acidic reaction environments, leading to the loss of active sites and poor cyclic stability [13,14,15,16]. Usually, the degradation efficiency will significantly decrease after 5–10 cycles of use, which makes it difficult to meet the practical application requirements. To address these pain points, researchers mostly modify iron-based amorphous alloys through element doping. Among them, Cu doping can accelerate the electron transfer of Fe^2+^/Fe^3+^ and improve catalytic activity [17], while Nb doping can enhance the stability of the amorphous structure and corrosion resistance, and inhibit excessive corrosion [18,19,20]. However, there are no systematic studies on the Cu and Nb dual synergistic modification of FeSiB-based amorphous alloys for MB catalytic degradation so far, and their dual synergistic catalytic mechanism, surface element evolution law, and structure–activity relationship are still unclear, leaving a gap in related research.

FeSiBCuNb is a typical Fe-based amorphous alloy with high structural stability, good corrosion resistance, and excellent catalytic activity [18,19]. The multi-component composition optimizes the amorphous forming ability and surface catalytic properties, making it a promising candidate for heterogeneous Fenton-like catalysis. As a new type of modified iron-based amorphous material, Fe_80_Si_6_B_10_Cu_1_Nb_3_ five-component amorphous alloy combines the catalytic activity of Fe, the corrosion resistance of Si, the amorphous forming ability of B, and the synergistic modification effect of Cu and Nb, which is expected to solve the problem that the activity and stability of traditional iron-based amorphous catalysts are difficult to balance. Based on this, this study took Fe_80_Si_6_B_10_Cu_1_Nb_3_ amorphous alloy as the catalyst to systematically investigate its catalytic degradation performance and kinetic characteristics for MB in the Fenton system, examine the effects of environmental factors such as temperature, pH, H_2_O_2_ concentration, and catalyst dosage on the degradation performance, determine the optimal reaction conditions, and evaluate its cyclic stability. The morphology, structure, and surface element evolution law of the catalyst before and after degradation were analyzed by SEM, XRD, and other characterization methods to clarify the Cu/Nb dual synergistic catalytic mechanism and the effect mechanism of surface element segregation on catalyst stability. The innovations of this study are as follows: breaking through the limitation of traditional single-element doping, constructing a Cu/Nb dual synergistic modified FeSiB-based five-component amorphous catalytic system to achieve the synchronous improvement of catalytic activity and cyclic stability; for the first time, clarifying the surface element segregation law of Fe_80_Si_6_B_10_Cu_1_Nb_3_ amorphous alloy during MB degradation; and establishing a new “corrosion-segregation-stability-synergistic catalysis” mechanism. The prepared catalyst can completely degrade MB within 9 min and maintain high efficiency after 20 cycles of use, which is significantly superior to the reported traditional iron-based amorphous catalysts. This study provides a new idea for the design and modification of high-performance heterogeneous Fenton catalysts, and also expands a new path for the practical application of iron-based amorphous alloys in the treatment of refractory dye-containing wastewater.

## 2. Results and Discussion

### 2.1. Degradation Properties of Fe_80_Si_6_B_10_Cu_1_Nb_3_

**Figure 1:**
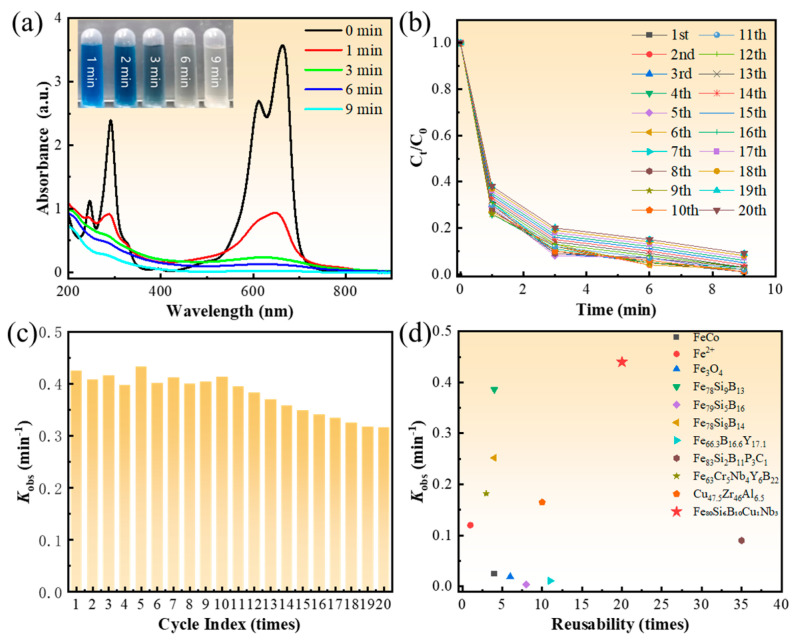
Performance characterization and literature comparison of Fe_80_Si_6_B_10_Cu_1_Nb_3_ amorphous alloy for MB degradation. (**a**) UV–Vis absorption spectra of MB solution at different degradation times (inset: digital photos of the solution at 1, 2, 3, 6, and 9 min); (**b**) time-dependent curves of relative MB concentration (*C*_t_/*C*_0_) during 20 consecutive cycles; (**c**) bar chart of *k*_obs_ over 20 cycles; (**d**) performance comparison of the as-prepared catalyst with other reported Fe-based/metal oxide catalysts (plot of *k*_obs_ vs. reusability times).

The catalytic performance of the Fe_80_Si_6_B_10_Cu_1_Nb_3_ amorphous alloy for MB degradation was comprehensively evaluated, as presented in Figure 1.

As shown in Figure 1a, the UV–Vis absorption spectra of the MB solution exhibit two characteristic peaks at 290 nm and 664 nm, corresponding to the aromatic ring and conjugated chromophore of MB molecules, respectively. The absorbance at 664 nm decreases rapidly with reaction time, dropping to nearly zero within 9 min, indicating complete destruction of the conjugated structure and full degradation of MB. The inset photographs visually confirm the decolorization process, where the deep-blue solution gradually fades to colorless, consistent with the spectral changes and reflecting the fast reaction kinetics. The methylene blue (MB) solutions before and after degradation were characterized via a total organic carbon (TOC) analyzer. The TOC removal efficiency of MB over the Fe_80_Si_6_B_10_Cu_1_Nb_3_ alloy reached 88.62%, demonstrating the excellent degradation performance toward MB. Figure 1b displays the time-dependent variation in relative MB concentration (*C*_t_/*C*_0_) during 20 consecutive cycles. All curves follow a pseudo-first-order kinetic trend, with *C*_t_/*C*_0_ decreasing sharply within the first 3 min and approaching zero at 9 min. Notably, the degradation profiles of the 11th to 20th cycles nearly overlap with the first run, demonstrating negligible loss of catalytic activity after repeated use and highlighting the excellent cyclic stability of the catalyst.

Figure 1c summarizes the *k*_obs_ across 20 cycles. The *k*_obs_ values remain stable at 0.40–0.45 min^−1^ for the first 10 cycles, reflecting a high and consistent initial catalytic activity. After 20 cycles, the *k*_obs_ only slightly decreases to 0.32–0.35 min^−1^, with a retention rate of over 70%, indicating a gentle and controllable deactivation process rather than catastrophic failure. This mild activity loss is attributed to the selective surface corrosion and elemental segregation observed in SEM/EDS analysis, rather than bulk crystallization or structural collapse. Figure 1d and Table 1 present a performance comparison with other reported catalysts, where the *x*-axis represents reusability times and the *y*-axis represents *k*_obs_. The Fe_80_Si_6_B_10_Cu_1_Nb_3_ catalyst achieves the highest *k*_obs_ (0.44 min^−1^) among all reference samples, while maintaining a reusability of 20 cycles, far exceeding most Fe-based amorphous alloys, metal oxides, and homogeneous Fenton catalysts [19,20,21,22,23,24,25,26]. Compared with other reported Fe-based amorphous catalysts [27,28,29,30,31,32,33,34,35], the FeSiBCuNb amorphous alloy prepared in this work exhibits superior catalytic performance for methylene blue degradation. It can achieve rapid and efficient removal of methylene blue under mild conditions, showing higher degradation efficiency and faster reaction rate within a shorter time. In addition, the catalyst maintains good structural stability and reusability during multiple cycles, which demonstrates its great potential in practical wastewater treatment.

This outstanding combination of high degradation rate and long cycle life underscores the significant advantages of the Cu-Nb synergistic modification and the great potential of the Fe_80_Si_6_B_10_Cu_1_Nb_3_ amorphous alloy for practical wastewater treatment applications.

**Figure 2:**
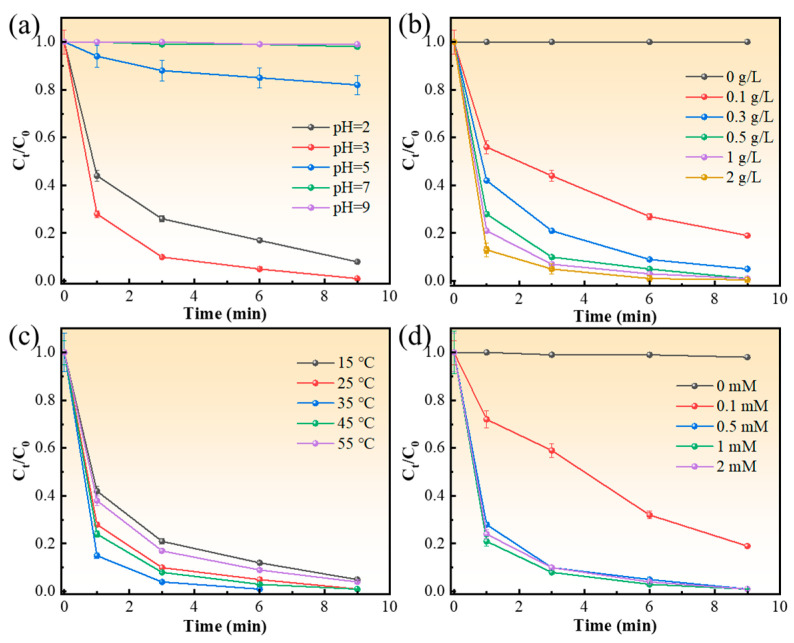
Single-factor optimization of MB degradation over Fe_80_Si_6_B_10_Cu_1_Nb_3_ amorphous alloy catalyst. (**a**) Effect of solution pH on MB degradation; (**b**) effect of catalyst dosage on MB degradation; (**c**) effect of reaction temperature on MB degradation; (**d**) effect of H_2_O_2_ concentration on MB degradation.

The effects of solution pH, catalyst dosage, reaction temperature, and H_2_O_2_ concentration on the catalytic degradation of MB over the Fe_80_Si_6_B_10_Cu_1_Nb_3_ amorphous alloy were systematically investigated, as presented in Figure 2.

As shown in Figure 2a, the degradation performance is strongly pH-dependent. The fastest degradation rate is achieved at pH 3, where the relative MB concentration (*C*_t_/*C*_0_) drops to nearly zero within 9 min. A slightly slower degradation rate is observed at pH 2, which is caused by the excessive protonation of surface Fe and Cu bimetallic active sites. Under strongly acidic conditions, abundant hydrogen ions occupy and cover the catalyst’s active centers, hindering H_2_O_2_ activation and hydroxyl radical generation, thereby reducing catalytic activity. In contrast, weakly acidic (pH 5–6) and neutral-to-weakly alkaline conditions (pH 7–9) significantly inhibit the Fenton reaction. Nearly no methylene blue degradation is achieved at pH 9, because alkaline environments promote the formation of inactive iron and copper hydroxide precipitates on the catalyst surface, which passivate the catalytically active surface and ultimately terminate the pollutant degradation reaction. Figure 2b displays the influence of catalyst dosage. Negligible MB degradation is observed in the absence of catalyst (0 g/L), confirming that the reaction is catalyzed by the Fe_80_Si_6_B_10_Cu_1_Nb_3_ alloy. Dosages of 0.1 g/L and 0.3 g/L result in incomplete degradation, while 0.5 g/L, 1 g/L, and 2 g/L all achieve nearly complete removal within 9 min, with no significant differences among them. This indicates that 0.5 g/L is the economically optimal dosage, providing sufficient active sites without unnecessary material consumption. The effect of reaction temperature is illustrated in Figure 2c. The degradation rate accelerates with increasing temperature from 15 °C to 55 °C, as higher temperatures promote the activation of H_2_O_2_ and the diffusion of reactants. Complete degradation is achieved within 9 min at 25 °C and above, and 25 °C is selected as the optimal temperature to balance reaction efficiency and energy consumption. Figure 2d shows the impact of H_2_O_2_ concentration. No obvious degradation occurs without H_2_O_2_ (0 mM), verifying the essential role of the Fenton-like reaction. A concentration of 0.1 mM leads to incomplete degradation, while 0.5 mM, 1 mM, and 2 mM all achieve nearly complete removal within 9 min. This suggests that 0.5 mM is sufficient to generate enough ·OH for MB oxidation, and excess H_2_O_2_ does not further enhance the performance due to radical scavenging effects.

### 2.2. Characterization Analysis of Fe_80_Si_6_B_10_Cu_1_Nb_3_ Before and After Degradation

**Figure 3:**
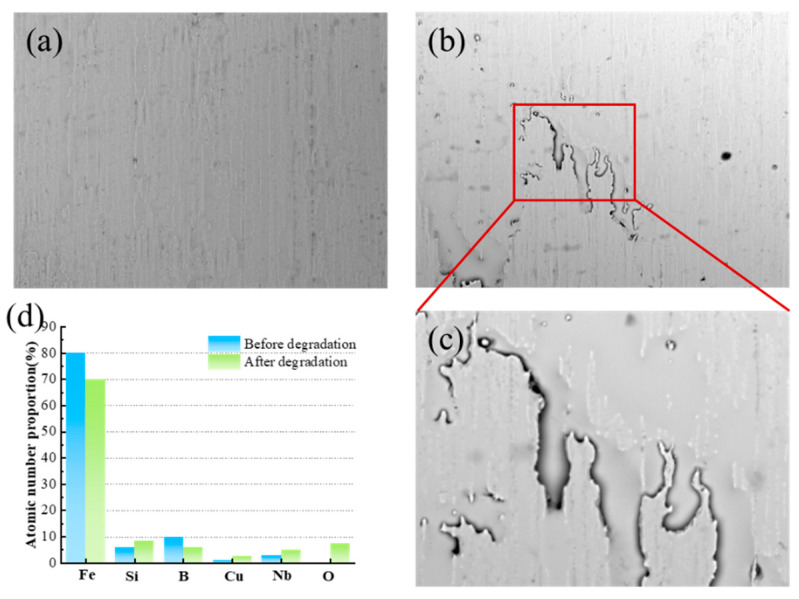
Morphology and surface elemental composition of Fe_80_Si_6_B_10_Cu_1_Nb_3_ amorphous alloy before and after degradation. (**a**) SEM image before degradation; (**b**) SEM image after degradation; (**c**) magnified SEM image of the red box in (**b**); (**d**) bar chart of atomic number proportions of surface elements before and after degradation.

The morphology and surface elemental evolution of the Fe_80_Si_6_B_10_Cu_1_Nb_3_ amorphous alloy before and after 20 consecutive degradation cycles were systematically characterized by scanning SEM and EDS, as presented in Figure 3.

As shown in Figure 3a, the fresh catalyst exhibits a smooth, dense, and homogeneous surface with only faint rolling streaks, reflecting the inherent structural uniformity and excellent corrosion resistance of the as-prepared amorphous alloy. In sharp contrast, after prolonged cyclic degradation, the surface undergoes pronounced selective corrosion, as visualized in Figure 3b and the magnified view in Figure 3c. Irregular, worm-like corrosion grooves and shallow pits are formed along the original rolling direction, indicating preferential dissolution of certain elemental regions. Notably, despite the localized corrosion, the overall structural integrity of the catalyst remains intact, with no signs of large-scale peeling, cracking, or crystallization, which is critical for maintaining stable catalytic performance over repeated cycles. The Brunauer–Emmett–Teller (BET) surface area of the FeSiBCuNb amorphous alloy catalyst was determined to be 12.76 m^2^/g, with a total pore volume of 0.048 cm^3^/g and an average pore diameter of 14.12 nm, indicating the mesoporous structure of the material according to the IUPAC classification standard.

EDS analysis (Figure 3d) further reveals the selective surface elemental segregation and leaching behavior during the degradation process. Compared to the fresh sample, the atomic proportion of Fe decreases slightly from 80% to 70%, confirming the controlled leaching of Fe ions into the solution to participate in the Fenton-like reaction. Similarly, B shows a minor loss from 10% to 5%, likely due to acidic dissolution. In contrast, significant enrichment is observed for Cu, Nb, Si, and O: the atomic fraction of Cu rises from 1% to 3%, suggesting surface segregation of Cu species that act as additional active sites to accelerate electron transfer and ·OH generation. Nb increases from 2% to 5%, forming dense NbOₓ passivation layers that effectively suppress excessive corrosion and preserve the amorphous matrix. Si is enriched from 5% to 8%, contributing to the formation of a SiO_2_-rich protective film. Meanwhile, the O content increases from nearly 0% to 8%, indicating the formation of metal oxides on the surface. This selective elemental evolution constructs a robust, self-passivating active interface, where Cu enhances catalytic activity while Nb and Si stabilize the structure, perfectly explaining the outstanding cyclic stability observed in the degradation experiments [18,19].

**Figure 4:**
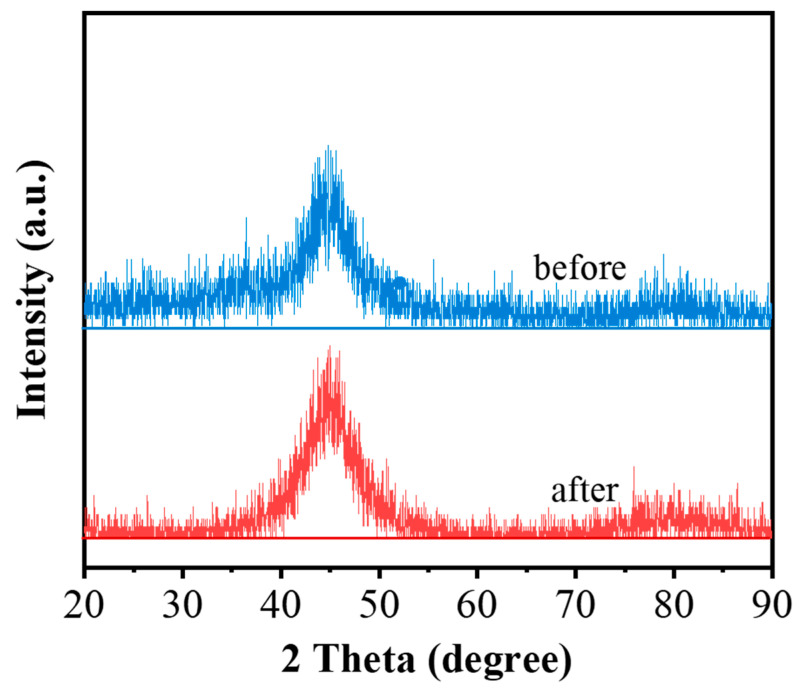
XRD patterns of Fe_80_Si_6_B_10_Cu_1_Nb_3_ amorphous alloy before and after degradation.

The XRD patterns of the Fe_80_Si_6_B_10_Cu_1_Nb_3_ amorphous alloy before and after 20 consecutive degradation cycles are presented in Figure 4. The XRD pattern of FeSiBCuNb amorphous alloy displays a broad and diffuse diffraction peak at 2θ = 40–50°, without any sharp crystalline peaks, which is the typical characteristic of Fe-based amorphous alloys reported in the previous literature [19,20]. This result confirms the successful preparation of a single amorphous structure without crystallization. After prolonged cyclic degradation, the position, width, and intensity of the diffuse peak remain nearly identical to the fresh sample, with no emergence of new diffraction peaks indicative of crystallization. This result confirms that the amorphous structure of the Fe_80_Si_6_B_10_Cu_1_Nb_3_ alloy is well preserved during the Fenton-like reaction, demonstrating exceptional structural stability. The retention of the amorphous state, combined with the surface self-passivation effect induced by Nb and Si enrichment, is responsible for the outstanding cyclic durability observed in the catalytic degradation experiments.

Combined with previously reported studies on Fe-based amorphous alloys, the loose and corroded surface microstructure can continuously expose fresh bimetallic (Fe-Cu) active sites during the Fenton-like reaction. Although further microscopic and valence characterization (TEM, XPS and elemental mapping) is not available in this work, the consistent SEM and XRD results sufficiently confirm the successful fabrication of amorphous FeSiBCuNb alloy and its structural stability during cyclic reactions. The surface corrosion behavior observed from SEM images reasonably explains the continuous activation of H_2_O_2_ and stable generation of hydroxyl radicals, verifying the reliable catalytic performance of the prepared amorphous alloy catalyst.

### 2.3. Mechanism Analysis of Fe_80_Si_6_B_10_Cu_1_Nb_3_ Catalytic Degradation of MB

**Figure 5:**
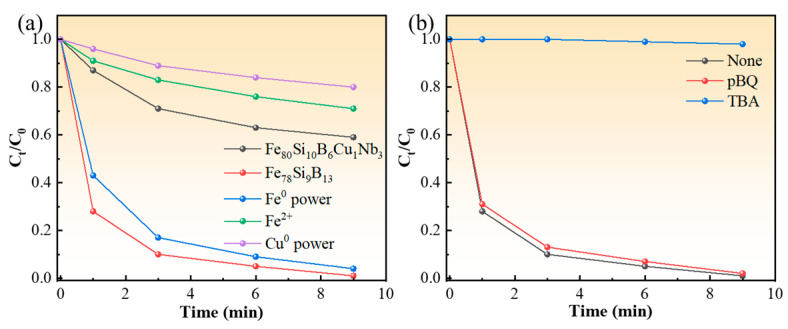
Catalytic activity comparison and radical quenching experiments of Fe_80_Si_10_B_6_Cu_1_Nb_3_ amorphous alloy. (**a**) Comparison of MB degradation performance over different catalytic systems; (**b**) effect of different quenchers on degradation (pBQ for ·O_2_^−^, TBA for ·OH).

The catalytic performance of the Fe_80_Si_10_B_6_Cu_1_Nb_3_ amorphous alloy was systematically evaluated by comparative analysis with other Fe-based catalytic systems and radical quenching experiments, as depicted in Figure 5a. The degradation kinetics of MB over four different Fe-based catalysts were compared. The Fe_80_Si_10_B_6_Cu_1_Nb_3_ amorphous alloy exhibits the highest catalytic activity, with the relative MB concentration (C_t_/C_0_) dropping rapidly to nearly zero within 9 min, clearly outperforming all other reference samples. The binary Fe_78_Si_9_B_13_ amorphous alloy shows slightly slower degradation kinetics, achieving complete MB removal only at the end of the 9 min test, which confirms that the incorporation of Cu and Nb plays a critical role in enhancing the catalytic efficiency. In sharp contrast, Fe^0^ powder and homogeneous Fe^2+^ ions display much lower activity: after 9 min, approximately 70% and 60% of MB still remain in the solution, respectively. The poor performance of Fe^0^ powder is attributed to its low specific surface area and sluggish electron transfer, while homogeneous Fe^2+^ suffers from rapid iron precipitation and limited reusability, highlighting the advantages of the heterogeneous Fe_80_Si_10_B_6_Cu_1_Nb_3_ amorphous alloy in terms of both activity and practical applicability. Single iron and copper components exhibit certain Fenton-like catalytic ability, but their degradation efficiency is relatively low. Zero-valent iron shows slightly higher activity than zero-valent copper. However, both of them are much less effective than FeSiBCuNb metallic glass. This demonstrates that the excellent catalytic performance originates from the Fe–Cu bimetallic synergistic effect, rather than the individual contribution of Fe or Cu alone.

Figure 5b presents the results of radical quenching experiments to identify the dominant reactive oxygen species (ROS) in the Fenton-like system. Three conditions were tested: a blank control, p-benzoquinone (pBQ, a specific scavenger for superoxide radicals ·O_2_^−^), and tert-butanol (TBA, a selective scavenger for hydroxyl radicals ·OH). The degradation profile with pBQ is nearly identical to the blank control, with only a negligible slowdown in reaction rate, indicating that ·O_2_^−^ contributes minimally to MB oxidation. Conversely, the addition of TBA almost completely suppresses the degradation reaction, with *C*_t_/*C*_0_ remaining at 1.0 throughout the entire 9 min period. This stark difference confirms that ·OH is the primary reactive species responsible for the oxidative degradation of MB. Combined with the EDS results showing Cu enrichment on the catalyst surface, it can be inferred that the surface-segregated Cu species accelerate the Fe^2+^/Fe^3+^ redox cycle, promoting the efficient generation of ·OH from H_2_O_2_ and thus leading to the superior catalytic activity observed.

**Figure 6:**
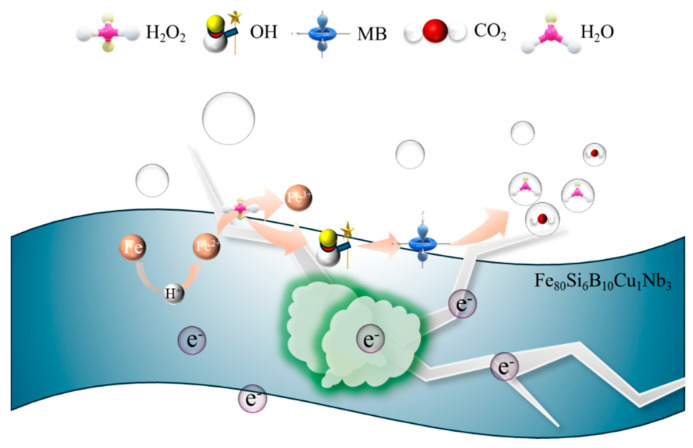
Schematic illustration of the Fenton-like reaction mechanism for MB degradation over Fe_80_Si_6_B_10_Cu_1_Nb_3_ amorphous alloy.

The detailed Fenton-like reaction mechanism for MB degradation over the Fe_80_Si_6_B_10_Cu_1_Nb_3_ amorphous alloy is illustrated in Figure 6, which integrates all experimental findings from catalytic performance, structural characterization, and radical identification.

The amorphous alloy matrix acts as a robust electron source, where Fe^0^ atoms donate electrons to generate Fe^2+^ ions in acidic solution, providing the initial active species for the Fenton reaction. The long-range disordered structure of the amorphous alloy ensures abundant undercoordinated Fe sites and efficient electron transfer, which is superior to crystalline Fe-based materials. The generated Fe^2+^ reacts with H_2_O_2_ to produce highly reactive ·OH radicals, which are responsible for the oxidative degradation of MB into CO_2_ and H_2_O. Notably, the surface-enriched Cu species serve as an efficient electron mediator, significantly accelerating the reduction of Fe^3+^ back to Fe^2+^, thereby sustaining the Fenton cycle and enhancing the generation of ·OH radicals. This Cu-mediated electron transfer explains the superior degradation kinetics observed in the experiments, outperforming binary Fe-based amorphous alloys and homogeneous Fe^2+^ systems [20]. The leaching concentration of Cu^2+^ from the FeSiBCuNb alloy is very low and meets the environmental discharge requirements, so the risk of secondary pollution is negligible.

Meanwhile, Nb and Si atoms segregate on the catalyst surface and form dense NbOₓ/SiO_2_ passivation layers during the reaction. These protective layers suppress excessive corrosion of the alloy matrix and control Fe leaching, preventing the formation of iron sludge and preserving the amorphous structure (as confirmed by XRD and SEM results). This self-passivation effect ensures the long-term cyclic stability of the catalyst, with negligible activity loss after 20 consecutive runs. The synergistic combination of Cu-enhanced catalytic activity and Nb/Si-mediated structural stability endows the Fe_80_Si_6_B_10_Cu_1_Nb_3_ amorphous alloy with both high degradation efficiency and excellent reusability, making it a promising candidate for practical wastewater treatment applications.

In summary, the reaction principle by which Fe_80_Si_10_B_6_Cu_1_Nb_3_ amorphous alloy catalyzes H_2_O_2_ to generate •OH and degrade phenol can be described as follows [18,19,20]:

Fe + 2H^+^ → Fe^2+^ + H_2_ (1)

Fe^2+^ + H_2_O_2_ → Fe^3+^ + •OH + OH^−^ (2)

Fe^3+^ + Cu^0^ → Fe^2+^ + Cu^2+^ (3)

MB + •OH → CO_2_ + H_2_O (4)

### 2.4. Future Perspectives

Although this study verifies the excellent performance of the Fe_80_Si_6_B_10_Cu_1_Nb_3_ amorphous alloy in simulated MB wastewater treatment, there are still some limitations: first, the experiment only focuses on a single dye pollutant, and the degradation effect in actual complex wastewater has not been investigated, so the substrate applicability needs to be expanded; second, the preparation cost of the catalyst is relatively high, and large-scale preparation has not been realized, which is difficult to meet the needs of industrial large-scale application; third, the dynamic characterization of interfacial electron transfer and active species generation during the reaction is not in-depth enough, and the mechanism research still needs to be improved; fourth, the catalyst activity decreases significantly under neutral and alkaline conditions, and its applicable pH range is narrow, which limits its practical application scenarios. Based on the above limitations, future work can be carried out in four directions: (1) expand substrate applicability, evaluate the degradation performance of the catalyst toward refractory organic pollutants such as phenols, antibiotics, and mixed dye systems, and explore the influence of coexisting ions on the catalytic effect; (2) optimize the preparation process, develop low-cost and large-scale preparation methods, and further improve the specific surface area, catalytic efficiency, and applicable pH range through nanocrystallization, porous structuring, or supporting modification; (3) conduct verification with real wastewater treatment and continuous-flow devices to evaluate the long-term operational stability, regeneration performance, and economic feasibility of the catalyst, promoting its engineering application; (4) employ in situ characterization techniques such as in situ Raman spectroscopy and in situ XPS to deeply reveal the dynamic mechanisms of interfacial structure evolution, electron transfer, and active species generation during the reaction, providing more solid theoretical support for the precise design and performance optimization of multi-component amorphous catalysts.

## 3. Experimental Materials and Methods

### 3.1. Experimental Apparatus

A UV-2600 UV–Vis spectrophotometer (Shimadzu, Kyoto, Japan) was used to measure the absorbance of methylene blue (MB) solution at 664 nm and calculate the corresponding dye degradation efficiency. An SU8010 scanning electron microscope (Hitachi, Tokyo, Japan) was utilized to characterize the surface morphology, corrosion characteristics, and microstructural evolution of the catalyst before and after the Fenton reaction. A D8 Advance X-ray diffractometer (Bruker, Karlsruhe, Germany) was employed to analyze the phase composition and structural stability of the amorphous alloy, as well as to monitor possible crystallization behavior during catalytic degradation. Auxiliary experimental instruments included a high-precision electronic balance (Sartorius, Göttingen, Germany), a pH meter (Mettler Toledo, Zurich, Switzerland), a constant-temperature water bath, a magnetic stirrer, an ultrasonic cleaner, a vacuum drying oven, a high-speed centrifuge, and 0.22 μm filter membranes. These devices were applied for sample weighing, pH adjustment, temperature control, sample pretreatment, drying, solid–liquid separation, and solution filtration, ensuring the accuracy, reproducibility and stability of all experimental data.

### 3.2. Degradation Experiment

The FeSiBCuNb amorphous alloy catalyst was prepared by a single-roller melt-spinning method. The pure metal elements (Fe, Si, B, Cu, Nb) were weighed according to the nominal atomic ratio and melted into an alloy ingot under an argon atmosphere. Then, the ingot was melted again and ejected onto a high-speed rotating copper roller to obtain continuous amorphous alloy ribbons. The as-spun ribbons were collected, crushed into small pieces, and used directly for catalytic experiments without further treatment. All chemicals were of analytical grade, and deionized water was used throughout the experiments. The alloy surface was polished to remove oxide layers, ultrasonically cleaned with deionized water and ethanol successively, and dried under vacuum at 60 °C before use. The MB solution was prepared as simulated wastewater, and H_2_SO_4_ and NaOH were used to adjust the initial pH to avoid interference of chloride ions with the iron-based catalytic system. The reaction temperature was controlled by a constant-temperature water bath. A certain amount of catalyst was added and stirred for 30 min to reach adsorption–desorption equilibrium, followed by the addition of H_2_O_2_ to initiate the Fenton reaction. Samples were collected at regular intervals, filtered through 0.22 μm membranes and measured by UV–Vis spectroscopy. The degradation rate was calculated according to the Lambert–Beer law, and pseudo-first-order kinetics were adopted to fit the reaction constants. The effects of temperature, pH, H_2_O_2_ concentration and catalyst dosage were systematically investigated to obtain optimal conditions. Twenty consecutive cyclic tests were performed, in which the catalyst was recovered by centrifugation, washed and dried after each run to evaluate reusability. Combined with SEM and XRD results, morphological evolution, elemental distribution and phase transformation were compared to reveal the Cu/Nb synergistic effect and amorphous stabilization mechanism.

To quantitatively evaluate the catalytic degradation performance of the Fe_80_Si_6_B_10_Cu_1_Nb_3_ amorphous alloy, the degradation curves were fitted with a pseudo-first-order kinetic model. The corresponding reaction rate constant was further calculated to assess the degradation rate toward MB. In the equation presented above, *C*_0_ and *C*ₜ denote the initial and instantaneous concentrations of the MB solution at time *t*, respectively; *k*_obs_ represents the observed kinetic reaction constant [36,37].

(5) $$C_{t}/C_{0}=\exp\left(-k_{\mathrm{obs}}t\right)$$

(6) $$k_{obs}=-In\left(\frac{C_{t}}{C_{0}}\right)/t$$

## 4. Conclusions

In this study, an Fe_80_Si_6_B_10_Cu_1_Nb_3_ quinary amorphous alloy was employed as a Fenton-like catalyst for the degradation of MB, and its catalytic performance, structural evolution, and reaction mechanism were systematically investigated. Under optimized conditions (pH = 3, catalyst dosage = 0.5 g/L, temperature = 25 °C, H_2_O_2_ concentration = 0.5 mM), the Fe_80_Si_6_B_10_Cu_1_Nb_3_ amorphous alloy achieves complete MB degradation within 9 min, with an apparent rate constant *k*_obs_ of 0.40–0.45 min^−1^, significantly outperforming Fe_78_Si_9_B_13_ amorphous alloy, pure Fe^0^ powder, and homogeneous Fe^2+^ systems. After 20 consecutive degradation cycles, *k*_obs_ only slightly decreases from 0.45 min^−1^ to 0.32 min^−1^ with negligible loss of degradation efficiency, demonstrating outstanding anti-deactivation ability and reusability. Structural characterization reveals that the bulk alloy remains amorphous after degradation, while selective elemental segregation occurs on the surface: slight leaching of Fe and B, accompanied by significant enrichment of Cu, Nb, Si, and O, forming a stable interface with Cu active sites and NbOₓ/SiO_2_ passivation layers. Mechanistic studies further confirm that surface-segregated Cu accelerates the Fe^2+^/Fe^3+^ redox cycle, promoting H_2_O_2_ activation to generate ·OH radicals (verified as the dominant reactive species by radical quenching experiments) and thus enhancing catalytic activity. Meanwhile, Nb and Si form dense passivation layers that suppress excessive corrosion and iron sludge formation while preserving the amorphous structure, achieving a synergistic balance between high activity and superior stability.

## Figures and Tables

**Table 1:** Comparison of reaction rate constants and number of cycles for different types of Fenton-like catalysts.

Materials	Organic Pollutants	K_obs_ (min^−1^)	Reusability	Ref.
FeCo	Acid Orange II	0.025	4	[21]
Fe^2+^	Rhodamin B	0.12	1	[22]
Fe_3_O_4_	Rhodamin B	0.019	6	[23]
Fe_78_Si_9_B_13_	Methyl Orange	0.386	4	[24]
Fe_79_Si_5_B_16_	Orange G	0.004	8	[19]
Fe_78_Si_8_B_14_	Orange II	0.252	4	[19]
Fe_66.3_B_16.6_Y_17.1_	Orange G	0.011	11	[19]
Fe_83_Si_2_B_11_P_3_C_1_	RhB	0.09	35	[25]
Fe_63_Cr_5_Nb_4_Y_6_B_22_	Methyl Blue	0.182	3	[20]
Cu_47.5_Zr_46_Al_6.5_	Acid Orange II	0.165	10	[26]
Fe_80_Si_6_B_10_Cu_1_Nb_3_	Methylene Blue	0.44	20	This work

## Data Availability

All data supporting the findings of this study are included in the published article.
